# Effects of the Layer Height and Exposure Energy on the Lateral Resolution of Zirconia Parts Printed by Lithography-Based Additive Manufacturing

**DOI:** 10.3390/ma13061317

**Published:** 2020-03-14

**Authors:** Laura Conti, Daniel Bienenstein, Mario Borlaf, Thomas Graule

**Affiliations:** Swiss Federal Laboratories for Materials Science and Technology, Laboratory for High Performance Ceramics, Empa, Ueberlandstrasse 129, 8600 Duebendorf, Switzerland

**Keywords:** additive manufacturing, resolution, ZrO_2_, LCM/DLP

## Abstract

Lithography-based ceramics manufacturing (LCM) processes enable the sophisticated 3 dimensional (3D) shaping of ceramics by additive manufacturing (AM). The build-up occurs, like many other AM processes, layer by layer, and is initiated by light. The built-in digital mirror device (DMD) enables the specific exposure of desired pixels for every layer, giving as a consequence a first estimation of the printing resolution in the x and y axes. In this work, a commercial zirconia slurry and the CeraFab 7500, both from Lithoz GmbH (Vienna, Austria), were used to investigate the potential of reaching this resolution. The results showed that the precision of a part is strongly dependent on the applied exposure energy. Higher exposure energies resulted in oversized dimensions of a part, whereas too low energy was not able to guarantee the formation of a stable part. Furthermore, the investigation of the layer thickness showed that the applied exposure energy (mJ/cm^2^) was acting in a volume, and the impact is visible in x, y, and z dimensions. The lowest applied exposure energy was 83 mJ/cm^2^ and showed the most accurate results for a layer thickness of 25 μm. With this energy, holes and gaps smaller than 500 μm could be printed; however, the measurements differed significantly from the dimensions defined in the design. Holes and gaps larger than 500 μm showed deviations smaller than 50 μm from the design and could be printed reliably. The thinnest printable gaps were between 100 and 200 μm. Concerning the wall thickness, the experiments were conducted to a height of 1 cm. Taking into account the stability and deformation of the walls as well, the best results after sintering were achieved with thicknesses of 200–300 μm.

## 1. Introduction

Additive manufacturing (AM) technology is an important area in all fields of materials science. Whereas many affordable products are available for polymers and metals, ceramic processes have only attracted intense attention recently. Ceramics are not used so widely in AM based on stereolithography as polymers and metals. The reason for this is the delicate post-processing after shaping, which includes drying, debinding, and sintering steps that give to the ceramics their characteristics and final shape. Nevertheless, some AM processes for shaping of ceramics and glasses have been developed. Direct shaping processes, such as selective laser sintering/melting (SLS/SLM) [1,2], are currently limited due to concerns over the quality of the parts and their surfaces. The majority of the AM processes for ceramics are indirect. For being able to shape a ceramic in indirect AM, a liquid phase (e.g., lithography-based ceramics manufacturing (LCM), stereolithograpy) or a meltable component (e.g., fused deposition modelling of ceramics (FDM) [3]) is required. The choice falls clearly to polymers, as they can later be burned out during the debinding process, or even be converted into a ceramic in the case of polymer derived ceramics (PDC). As binder combustion is associated with an increase of pressure and volume within the part, their utilization restricts the size for printed parts. In the case of the LCM process, this size limitation is 3 mm of thickness [4]. Over 3 mm, the debinding will be prolonged by a significant amount of time [4], and is therefore not recommended. Based on *German’s debinding theory* [5], the debinding time and the component’s size have a direct square law relationship; i.e., a part with double thickness will need four times more time to complete the debinding. For this reason, LCM printing is the process that is preferably being used for the production of small, precise parts, which is the focus of this study. Chen et al. [6] and Zocca et al. [7] listed a comparison regarding the resolution of AM processes for ceramics, wherein the slurry-based methods were those with the best resolution.

**Figure 1:**
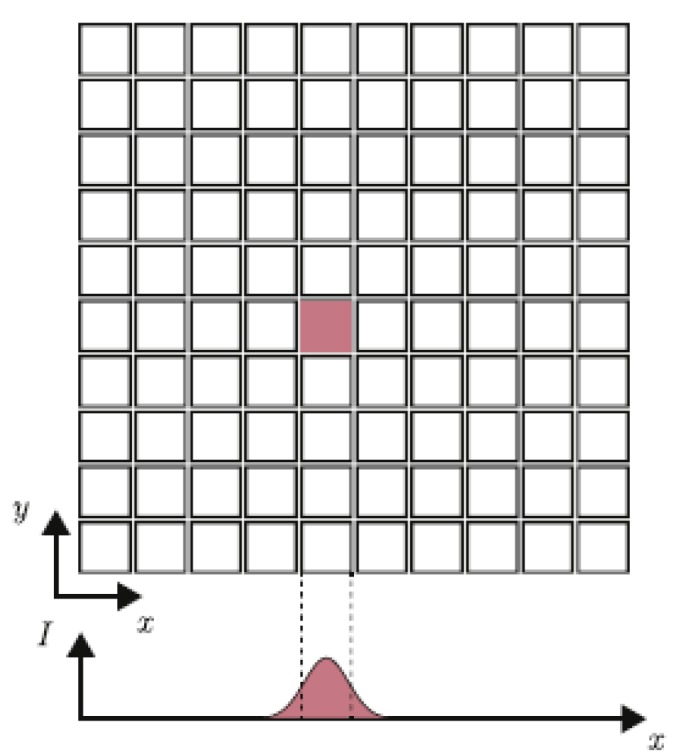
Irradiation profile of a single pixel adopted from [12].

AM by the LCM technique is based on light initiated polymerization of liquid mono-/oligomers to solid polymers to achieve a layer by layer build up. The process has been described in various papers and different systems [8,9,10], but none of them showed a detailed study on their resolutions. A first indication is given by the pixel size of the implemented digital mirror device (DMD). When looking at the irradiation profile of a single pixel, the intensity follows a Gaussian distribution [11], as shown in Figure 1.

This phenomenon is irrelevant if the neighboring pixels are irradiated as well. In the case of the part edges, exceeding of the pixel boundaries causes an inaccuracy of the generated specimen compared to the CAD (computer aided design) [12]. This problem is often referred to as cross–talk effect [11] and shows in so-called over-polymerization of a printed part. Furthermore, the irradiated light is attenuated by the absorption and scattering effects due to the ceramic particles. The light-absorption, as defined by the Beer–Lambert Law (Equation (1)), affects the penetration of the light and therefore the cured depth in the z direction.

(1) $$E_{p}=D_{p}\ln\left(\frac{E_{0}}{E_{c}}\right)$$

The polymerizations’ critical energy value (*E_c_*) has to be reached to start the curing of the polymer (*E_p_*). The *D_p_* inside the equation stands for the cure depth and the E_0_ for the energy radiating into the system. As soon as the critical energy value *E_c_* is reached, a chain of reactions is initiated by the radical photo polymerization [13].

The scattering leads to less focused light and the initialization of reactions in the x and y-axes, which results in a broadening of the exposed area [14]. This scattering occurs on the surface of the ceramic particles within the slurry and can be reduced by choosing ceramics and liquid phases with a small mismatch between their refractive indices [15,16]. Therefore, a higher solid loading of the ceramic slurry will enhance this scattering effect and result in broadening of the cured width and a reduction of the depth [14,17].

The material of choice for this study was zirconia because of its properties, including biocompatibility, high strength, small grain size after sintering, good chemical resistance, and high fracture toughness. 

Resolutions reported by other studies: Schwentenwein et al. and Harrer et al. reported strut thicknesses down to 200 μm [18] and 270 μm [19] on zirconia parts. Further, they reported 125 μm for walls or struts and 200 μm for defined pores or channels [8] in alumina parts. In the case of the 125 μm walls, the shape differed a lot from the experiments conducted herein. The printed piece (turbine blade) was curved and only approximately 6 mm in height. The already integrated curve in the blade makes it difficult to judge the deformation if any occurred. The alumina dispersion [20] shows a four times lower viscosity at 20 °C compared to zirconia [21]. Other studies also reported pore sizes down to 300 μm [16]. In this case, all the tested dispersions also showed lower viscosities (below 20 Pa*s) than the herein processed dispersion. A dilution of the given slurry was not an option, as its solid loading of 39 vol% is already the minimum when requesting to sinter dense parts after printing. Other AM authors report open pores from 0.8 mm and larger [22] and 400 μm after sintering [23] for 3 dimensional printing (3DP).

## 2. Experimental

### 2.1. Lithography-based Ceramics Manufacturing (LCM)

The shaping process was conducted with a customized CeraFab 7500 printer by Lithoz GmbH in Vienna, Austria, and has been described in several publications [1,6,10,11,12,18,24,25,26]. The customization concerns the device’s ability for VIS and UV polymerization. The UV polymerization is used for the development of formulations [25,27]. In this study, the commercial VIS unit was used as the dispersion provided by Lithoz calls for a wavelength of 460 nm. The pixel size is 40 μm in this case. The dispersion used was a zirconium-oxide-based dispersion with a solids loading of 39 vol.%. The BET (Brunauer-Emmet-Teller) surface was 5.2 m^2^/g and was measured with a Sorptionsanlage Coulter SA3100 by Beckman Coulter in California, USA after acrylate binder removal. The recommended exposure energy for the printing process is 110–190 mJ/cm^2^ according to the supplier. The studied values were in this range, but additional ones of 83 and 105 mJ/cm^2^ were also tested in order to find the best experimental parameters. These energy doses were reached by exposing the slurry with maximum intensity of 83.12 mW/cm^2^. 

### 2.2. Determination of Geometrical Restrictions Concerning Pore Size and Wall Thickness

**Figure 2:**
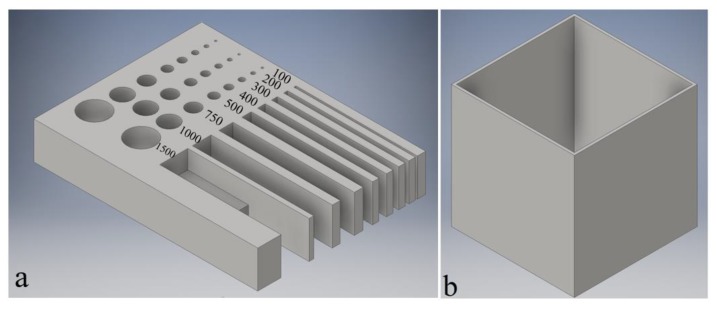
Computer aided design (CAD) models for the investigations of the diameters of holes (values in µm), the widths of gaps, and (**a**) the thicknesses of walls (**b**).

As already mentioned before, the LCM process is particularly suited for small parts with delicate details. In this study, three types of elements were investigated: the diameters of holes, the widths of gaps, and the thicknesses of walls. The first two of these were incorporated into one test component which is shown in Figure 2a and is referred to as a resolution plate. The diameter of holes and the width of gaps were chosen in a range of 0.1–1.5 mm. The thickness of the plate is 2 mm, enabling a rather fast printing time of 1.5 h when printing with a layer thickness of 25 μm (3 h for 10 μm and 45 min for 50 μm layer thickness). The reason why the investigation of the wall thicknesses was not incorporated into the resolution plate was to check the stability of walls in terms of their thickness. The thinner a wall, the less stable it will become in height. Therefore, the investigation of the wall thickness was conducted on cube cups 1 cm high and is shown exemplarily in Figure 2b for a 100 μm wall thickness. The investigations of the wall thicknesses were conducted with the thicknesses of 75, 100, 200, 300, 400, and 500 µm. 

The parts shown in Figure 2 were printed in two sets: the first set without any shrinkage compensation to investigate the printing error and the second set with a shrinkage compensation to determine the smallest dimensions produced for a sintered part. A shrinkage compensation of 1.354 in x and y directions was applied to the second set.

The cleaning of all printed parts was performed in the CeraCleaning station and using the commercial solution LithaSol 20 (Lithoz GmbH in Vienna, Austria). The measurement of the dimensions was performed by light microscopy (SteREO Discovery.V20” from Zeiss, Germany) and image analysis software (IMS Client). 

The measurements were carried out on several parts and locations. The calculation of the average of each different element is shown in Equation (2).

(2) $$\bar{x\;}=\frac{1}{n}*\overset{n}{\underset{i=1}{\sum }}a_{i}$$

where

$a_{i}$ = the values measured by light microscopy;
*n* = 3 for walls, gaps, and holes < 1.5 mm (CAD value);
*n* = 2 for ≥ 1.5 mm (CAD value);
$\bar{x}$ = average.

The calculation of the deviation (*dev*) of a given CAD value can be seen in Equation (3).

(3) $$dev=\bar{x}-x_{CAD}$$

where

$x_{CAD}$ = dimension of the CAD model

Therefore, if *dev* > 0, the measured element is too big compared to the CAD dimension, and thus a positive deviation to the value results. If *dev* < 0, the printed element is smaller/narrower in respect to the designed model. 

### 2.3. Thermal Post-Processing

**Figure 3:**
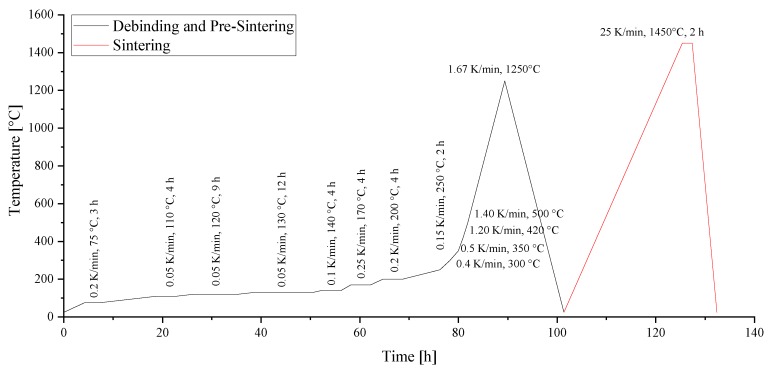
Debinding and sintering profiles.

The thermal post processing suggested by the supplier consists of one heat treatment for debinding and sintering. However, the program was divided into two separate steps, as the debinding and pre-sintering were not carried out in the same furnace as the sintering (Figure 3). 

The shrinkage of the parts was measured by thermo mechanical analysis on a Netzsch TMA 402 F3 (Netzsch Gerätebau GmbH in Selb, Germany), obtaining 11% after debinding and 29% after sintering at 1450 °C for 2 h. The detected shrinkage by TMA corresponded well with the suggested compensation of 1.354 by Lithoz GmbH.

## 3. Results and Discussion

### 3.1. Energy Related Resolution

**Figure 4:**
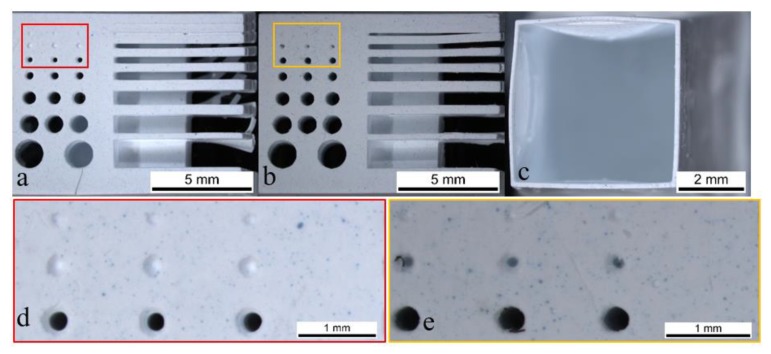
A resolution plate printed with 105 (**a**,**d**) and 83 (**b**,**e**) mJ/cm^2^ and a cube cup of 0.1 mm wall thickness (**c**) printed using 105 mJ/cm^2^ of energy.

Green parts: As already stated by Mitteramskogler et al. [24] and Ozog et al. [26], the dimensional accuracy strongly depends on the exposure energy applied. For the energy-related resolution, the suggested values were investigated, and values were below those recommended. These experiments were carried out with a layer thickness of 25 μm. The green resolution plates (Set 1) for 105 and 83 mJ/cm^2^ can be seen in Figure 4a,b. The smallest printable cube cup with 105 mJ/cm^2^ is shown in Figure 4c.

**Figure 5:**
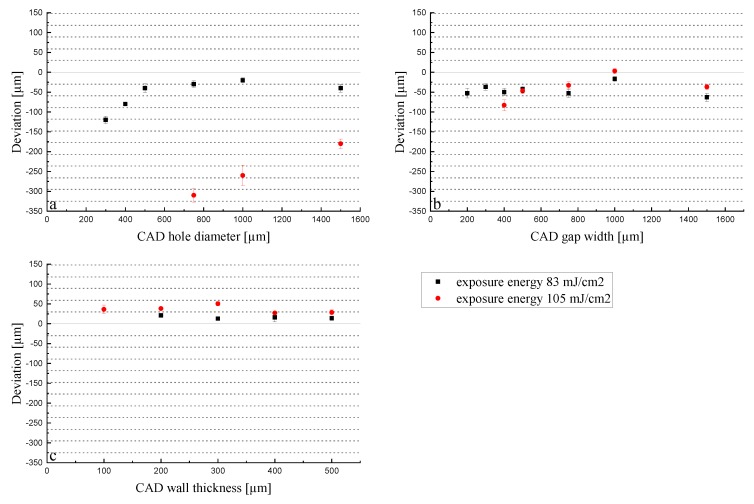
Deviations of the dimensions for holes (**a**), gaps (**b**), and walls (**c**) in terms of energy dose of green parts with respect to the CAD designs.

A difference concerning the printability of holes and gaps can already be seen when comparing Figure 4a,b. In the case of 105 mJ/cm^2^ there are only indications where the 0.3 mm holes should be present, whereas in the case of 83 mJ/cm^2^ the holes even go through the whole body (highlighted in the red and yellow rectangle and visualized in Figure 4d,e). This phenomenon can be explained by the scattering of light, which leads to a broadening of the exposed area, as explained in the introduction (Figure 1). Reactions in x and y directions are initiated, leading to an over-polymerization, and therefore to an over-sized part, which with holes and gaps makes the element smaller than anticipated. In the case of holes being present, this effect is even amplified by capillary forces that will trap the slurry in a hole instead of overcoming gravity forces and flowing out. Moreover, it should be taken into account that the scattering of the particles can enhance this over-polymerization effect, decreasing the hole/gap size and increasing the wall thickness. It is important to highlight that even if the used energy is lower than the recommended range by the supplier, the best results were obtained at 83 mJ/cm^2^; it is possible to print parts with holes with 0.3 mm diameters and gaps 0.2 mm in width. In the case of the walls, the problem mentioned before about their stability can be seen in Figure 4c. The 100 μm cube cup (CAD value) could be printed but the top view indicates a deformation of two of the four walls, attributable to the build-up process. The very thin and fragile walls are pushed into a high viscous slurry and drawn out of it again several times. After the process, these thin walls could be bended by little manual force and the cleaning process, which was rather challenging because the cups walls tend to flap during the air-supported cleaning. Additionally, the thinner the wall, the smaller the contact area between layers that ensures their adhesion. The thinnest printable wall was of 100 μm because the 75 μm one (CAD values) could not be printed, as the layers delaminated during the printing process, leading to a damaged part. A way to counter this problem would be to target a greater stability for the green part, which could be achieved by, e.g., a different reactive binder or a higher content of cross linker. As this investigation was conducted on a commercial product, the authors did not consider these changes. The deviations of the measured dimensions of the green parts from the CAD design are shown in Figure 5.

The absolute deviation for holes is bigger than the ones for gaps and walls, but gets smaller as the diameter increases. Regarding gaps and walls, there is not a clear trend, and this effect was not observed; the deviation remained unaffected by the size set in the CAD model. Moreover, the absolute deviation for all experiments increases with a bigger energy dose, although in the gaps this effect is not so clear; minimal differences could be found, and only below 500 µm. Contrary to holes and gaps, walls turned out larger than the CAD, which is given by the positive deviation. This is in agreement with the scattering effect, as in the case of holes and gaps, the dimensions of the non-polymerized area are of interest (smaller because of the scattering and due to that, negative deviation), whereas in case of the walls, the polymerized material is relevant (the scattering of the light promotes the polymerization of neighboring pixels enlarging the dimensions, leading to a positive deviation). In the case of holes and walls, the lower energy dose of 83 mJ/cm^2^ leads to a significantly lower deviation. Moreover, the deviations in Figure 5 often correlate with the pixel size of 40 μm (dotted lines). This trend is especially visible in the diameters of holes printed with 83 mJ/cm^2^. This indicates that the software has the tendency to expose pixels where only a fraction of the slice should be exposed. This results in a very regular deviation for the lowest exposure energy of 83 mJ/cm^2^. When observing, e.g., Figure 5c, the 100 µm wall thickness exposed with 105 mJ/cm^2^ resulted in a thickness of 136 µm. This indicates that not only was 120 µm (namely 3 pixels in width) exposed, but a certain amount of over-polymerization occurred as well. Following this logic, the 200 µm wall thickness should have a smaller deviation because it divides evenly with the 40 µm pixel size, but again we observe a similar deviation as for the 100 µm wall thickness (238 µm with 105 mJ/cm^2^). These errors can be explained by the position of the CAD model in the print file and the pixels. A part edge does not necessarily fall on a pixel border. In this case, the software has to decide whether to expose a pixel or not. If the pixel is exposed, the part is already produced larger without even considering the above-mentioned cross-talk and scattering effects that will lead to bigger parts. 

When the ranges of the deviations (x-axes) in Figure 5 are compared, the error of the capillary effect can be observed in case of the holes. This effect strongly affects the printability of smaller holes, as the capillary force overpowers the gravitational force of the slurry. As a result, the slurry remains in the tube instead of flowing out. Later, the fabrication of further layers may also polymerize the liquid that remained in the tube and therefore close it even more and leading to big deviation to the desired diameter. Additionally, larger holes tend to show a smaller deviation with increasing size, which is also in good agreement with the capillary rise of liquids in columns with wider diameters. Therefore, the larger the hole, the smaller the relative and absolute deviation from the CAD design. In the case of the exposure energy of 83 mJ/cm^2^, the deviations converge to a value of about −50 μm for 500 μm holes and larger. This convergence is less clear for gaps, as the capillary effect is not affecting the smaller gaps, as in case of the holes. As expected, there is no convergence for walls.

**Figure 6:**
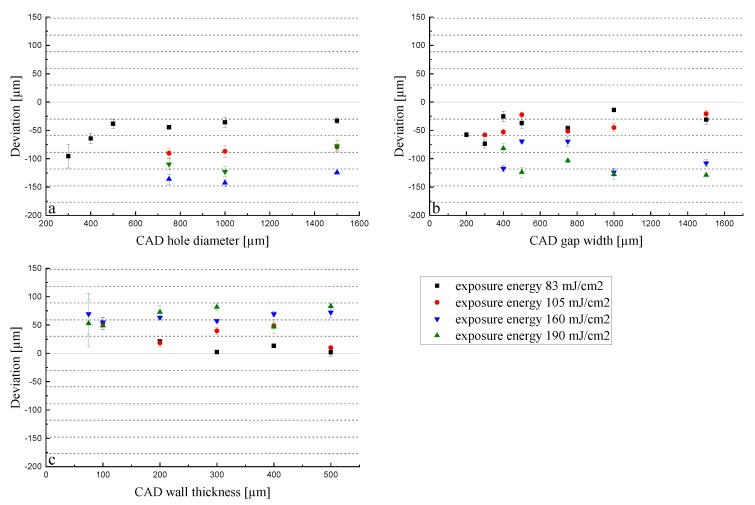
Deviation of the dimensions of sintered parts for holes (**a**), gaps (**b**), and walls (**c**) in terms of energy dose with respect to the CAD model.

Sintered parts: The parts with shrinkage compensation (Set 2) were printed with the energies of 83, 105, 160, and 190 mJ/cm^2^ to investigate the energy area suggested by the supplier. The deviations of the measured dimensions for the sintered parts are shown in Figure 6.

For all conducted experiments it is apparent that the absolute deviation increases with energy dose. The suggested values of 160 and 190 mJ/cm^2^ resulted in a large deviation across all diameters, gaps, and thicknesses, indicating that the over-polymerization effect is more predominant in comparison to the capillary effect. Smaller walls down to 150 μm could be printed, but a larger deviation from the desired value was observed. Contrary to holes, this deviation did not increase as significantly with higher exposure energy but is still visible. This is attributable to the space between the individual elements. In the case of holes and gaps, the small distance between exposed pixels enhances polymerization inside the hole/gap, whereas in the case of walls, the cups were positioned 6 mm apart, and after approximately 80 μm (Figure 6c) from the part’s edge, the reactions stop. This is related to the mobility of the free radicals and the attenuation of light. The mobility of the free radicals is restricted more and more by the rise of viscosity and results in a quick drop of the polymerization. Further, the light is attenuated by absorption, so reactions are not initiated as the probability of the photoinitiator being activated by light significantly lowers. The deviation of the printed holes (Figure 6a) is not dependent on the diameter, as can also be seen in the green bodies (Figure 5a, from a 500 µm diameter). This could be explained by the residues of slurry in the smaller holes of the green part, as the cleaning is a critical point of the procedure and should be treated as such. A more intense cleaning of the printed parts by, e.g., soaking the part in the cleaning solution (LithaSol 20) was evaluated and resulted in damaging the parts by means of layer delamination, and was therefore excluded.

### 3.2. Layer-Thickness-Related Resolution

**Figure 7:**
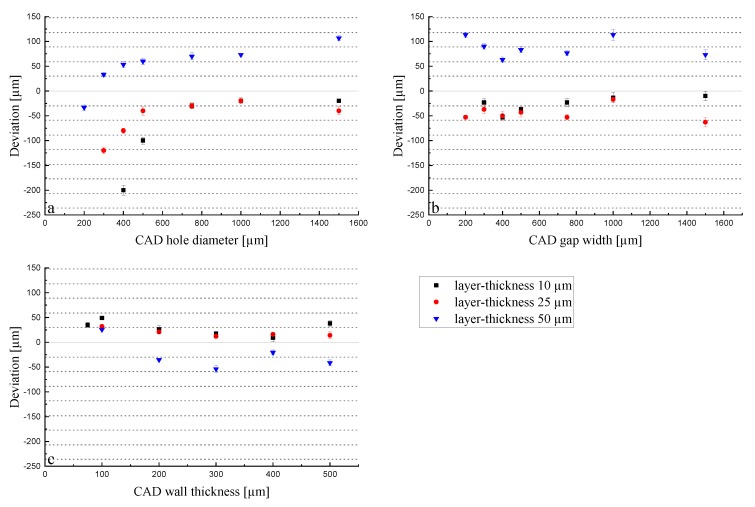
Deviation of the dimensions for holes (**a**), gaps (**b**), and walls (**c**) in terms of layer thicknesses of green parts with an exposure energy of 83 mJ/cm^2^.

Green parts: When looking further into the LCM process, it becomes clear that the initiated polymerization is not only taking place in x and y dimensions but also in z dimension, which is the key element for a 3D build up. Thus, when applying a certain energy dose, the unit is mJ/cm^2^, but it actually occurs in a certain volume, as is given by the Lambert–Beer Law (see Equation (1)). An exemplarily investigation is the measurement of the cure depth. The cure depth measures the cured thickness in a pool of reactive slurry when applying a defined energy. The measured cure depth for an energy dose of 83 mJ/cm^2^ amounts to 81.1 µm ± 2.5 µm (n = 5). Thus, when limiting the process to a certain layer height, the reactions come to a halt in z dimension but not in x and y dimensions, where enough liquid slurry still remains. To ensure a stable build-up, usually, the cure depth needs to be higher than the applied layer thickness to ensure enough adhesion between the layers. When working with a layer thickness of 25 µm, the factor between layer thickness and cure depth amounts to 3.2 (8.1 for 10 µm layer thickness and 1.6 for 50 µm layer thickness). The conducted experiments help to understand the volume effect when always applying the same energy dose (83 mJ/cm^2^) to different layer thicknesses, and thus, volumes. In Figure 7, the deviations for the holes, gaps, and walls are shown for layer thicknesses of 10, 25, and 50 μm in the green body (set1). 

**Figure 8:**
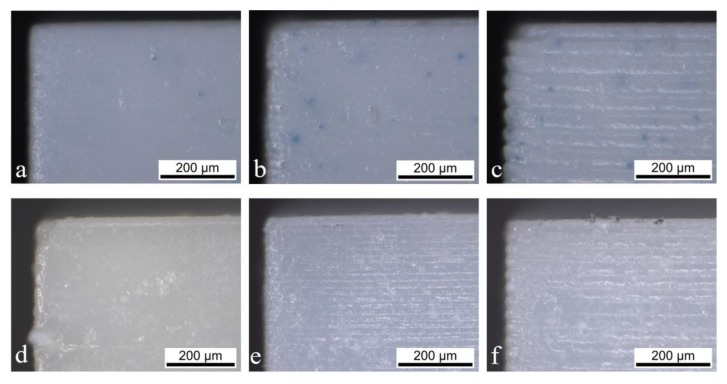
Side view of the green bodies printed with 10 (**a**), 25 (**b**), and 50 μm (**c**) layer thicknesses and the sintered bodies of 10 (**d**), 25 (**e**), and 50 μm (**f**) thicknesses.

For a layer thickness of 10 and 25 μm, the deviations do not differ so much from each other in comparison to the 50 μm layer thickness. The deviation of holes converges to a minimum while gaps and walls do not show this behavior. In all cases, they are in good agreement with the previously stated volume effect. As in the case of the 10 μm layer thickness, the exposure energy of 83 mJ/cm^2^ is acting on a five-times-smaller volume compared to the 50 μm layer thickness samples. This provokes the over-polymerization effect, leading to smaller holes and gaps and thicker walls. This difference in z (thickness) impacts the accuracy in the x-y plane, as the polymerization occurs in the three dimensions, and therefore, diffuses in the lateral directions. The holes (Figure 7a) showed a similar trend when different energies were applied (Figure 5a). They converge to an optimum that starts from holes larger than 500 μm. For the 10 and 25 μm layer thicknesses, the deviation remains around −40 μm, and for the 50 μm layer thickness, a positive deviation was obtained for all sizes except for 200 μm (−40 μm). This means that the applied exposure energy of 83 mJ/cm^2^ is not enough to print the part according to the CAD drawing, which can also be explained by the cure depth of the slurry used. As the factor between cure depth and layer thickness only amounts to 1.6 for a layer height of 50 µm, the initiated reactions lead to insufficient polymerization of the slurry during the process. This could also be related to the absorption effect: the larger the volume, the higher the absorption. On the other hand, this enables the possibility of fabricating holes (in a piece, in tubes, in pores, etc.) with a very small diameter (150 μm) due to the −30 μm deviation from the 200 μm set hole diameter. The low energy for the print with a 50 μm layer thickness is also visible in the case of gaps (Figure 7b) with the same trend as in the holes. In that case, the deviation does not change with the gap width but remains in the range of 50–100 μm from the real value for all printed gaps. This is again attributable to the capillary effect that occurs in the holes but not in the gaps. In the investigation of the green walls, the same trend was observed. The energy of 83 mJ/cm^2^ was not high enough for the 50 μm layer thickness, so the walls were printed too thin, which is displayed by a negative deviation from the target value (Figure 7c). The samples with 10 and 25 μm layer thicknesses showed, again, a very similar behavior in the deviation. Taking into account all the graphs, the reduction of the layer thickness from 25 to 10 μm does not influence the lateral resolution significantly. As a result, this reduction is unnecessary from a temporal point of view, as the printing time is doubled for 10 μm compared to 25 μm. The increase of the layer thickness to 50 μm not only results in a faster printing job, but also enables the fabrication of thinner walls and smaller holes, as can be observed in Figure 7. Nevertheless, the big deviation from the target value should be considered when printing with these parameters. These results only concern the lateral resolution; the thickness related dimension should also be taken into consideration when changing the layer thickness. While a significant amount of time can be saved by increasing the layer thickness to 50 μm, the resolution in z direction suffers significantly (Figure 8).

In the case of a layer thickness of 10 μm (Figure 8a), the formation of the layer structure can barely be observed, whereas a layer thickness of 50 μm shows clear steps and a laminated structure. Thus, the surface quality of the part is significantly impacted by this change and even leads to delamination in the case of very thin walls of up to 300 μm thickness. Thus, depending on the application, it makes sense to change the layer thickness to higher values to have a faster sample production or to reduce it to improve the surface finish in z direction. The more impactful action to work against this effect would be to increase the energy dose when printing with a higher layer thickness. By increasing the energy dose, the cured depth in z direction is increased and a more homogeneous adhesion between layers can be achieved. As the manufacturer’s recommendation of the energy exposure represents a range of 110–190 mJ/cm^2^, there is room for improvement concerning this visible difference in layer thickness. While the sintering (Figure 8d–f) of the bodies reduces the visibility of this effect, it can still be seen clearly.

**Figure 9:**
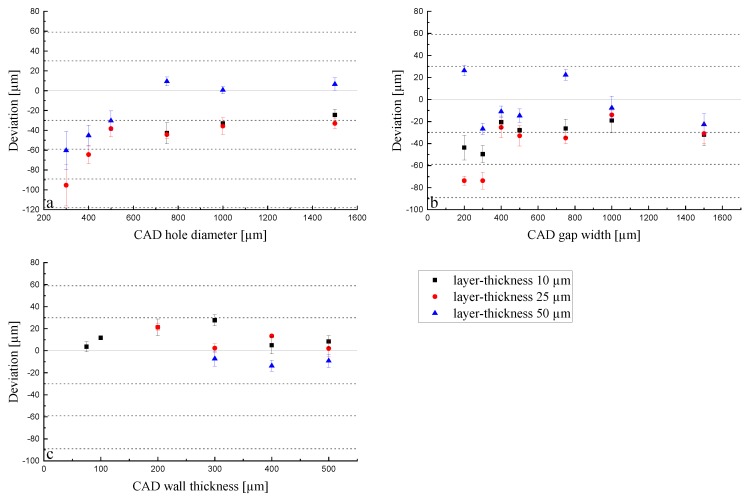
Deviation of the dimensions for holes (**a**), gaps (**b**), and walls (**c**) in terms of layer thicknesses of sintered parts with an exposure energy of 83 mJ/cm^2^.

Sintered parts: The parts with shrinkage compensation (Set 2) were printed with the same layer thicknesses as before. The deviation of the sintered parts can be seen in Figure 9.

Holes under 750 μm could not be printed for a layer thickness of 10 μm. This can be explained by a broadening of the exposed area, as the reaction was inhibited in z direction by the limitation of the layer height but not in x and y directions. Holes under 500 μm could not be printed with a 10 µm layer thickness; however, thin gaps were possible, with a large deviation from the target dimension, however. The trends do not differ too much from each other in comparison to the results obtained in Figure 7. Moreover, the volume effect is visible in all plots, showing, as before, that the dimensions of the 10 and 25 μm layer thickness prints are similar, while there is a clear difference visible in the case of the 50 μm layer thickness for holes. Regarding holes, an exposure energy of 83 mJ/cm^2^ on a 50 μm layer thickness led to the lowest deviation; thus, the most accurate dimensions compared to the CAD design were obtained. The smallest printable hole had a target diameter of 500 μm, measuring 470 μm after sintering. The plots of the gap widths and wall thicknesses do not show results as clear as before, but they are still in good agreement with the previous results shown in this section. Nevertheless, the influences of the volume and cure depth can be seen in the experiments for the wall thickness. Some walls (< 300 µm CAD thickness) could not be printed, as they were not stable enough due to insufficient curing and polymerization and green body strength.

## 4. Conclusions

This study highlights the major resolution limitations of the LCM process. The achieved resolutions are comparable to reported values with similar systems in the literature. While only mentioning the resolution in the lateral dimension, the layer height showed a noteworthy impact on these values. The use of a standardized, simple computer aided design enabled us to make meaningful comparisons in this study and could be used by future scientists on their parts. The investigated models focus on often-implemented features in complex parts that are error-prone in additive manufacturing. While many known influences on the LCM process could have been proven, the study also shows the direct impacts on the quality and accuracy of the part.

### 4.1. Precision of Green Parts (Set 1)

Holes down to 200 μm could be printed, but the investigation of the deviation shows that holes smaller than 500 μm in diameter exhibit a big deviation from the set dimension. The deviation of gap widths did not show a clear correlation to the actual width—whereas holes did—and the thinnest one had a width of 150 μm. Regardless of exposure energy or layer thickness, the thinnest stable walls had thicknesses between 300 and 400 μm in the green state.

### 4.2. Precision of Sintered Parts (Set 2)

The results from the sintered parts are in agreement with the values for green bodies. The smallest printable holes had diameters between 400 and 500 μm. Even though smaller diameters could be achieved in the green state, the measurements of these holes showed that they closed during the sintering and could therefore not be counted as holes anymore. The smallest printed gaps were about 200 μm, which due to over-polymerization, resulted in widths between 110 and 170 μm. The thinnest sintered walls were under 100 μm; however, as in the case of the green walls, they exhibited deformation because of the height. This deformation was not visible anymore from a thickness of 200–300 μm, which is in good agreement with the above-mentioned thickness of 300–400 μm in the green state.

## 5. Outlook

This study showed an investigation of the two most impactful processing parameters on the additive manufacturing of ceramics by digital light processing. While it could be shown that the exposure energy and the layer thickness have big influences on the printability and on the quality of a part, another parameter could also be identified as crucial to the shaping process. This parameter is the composition of the slurry, which was given in this study. To gather further knowledge on how the reactive slurry can influence the part, more studies are necessary. For example, the authors expect a lower deviation and therefore higher precision for parts printed with slurries with lower solid loading. This is due to the often-stated scattering of light in the interface between particle and liquid phases of the slurry. The consequences of a lower solid content will be, most likely, longer debinding and lower density of the sintered part. Another point that was stated during the discussion of the results regarded the types of reactive binders and their functionalities. A higher functionality (3+) of the reactive binders would increase the green body strength by means of cross-linking and be beneficial for the printing of thin walls and studs.

These are further approaches that the authors would like to point out as crucial and of high significance for lithography-based additive manufacturing.
